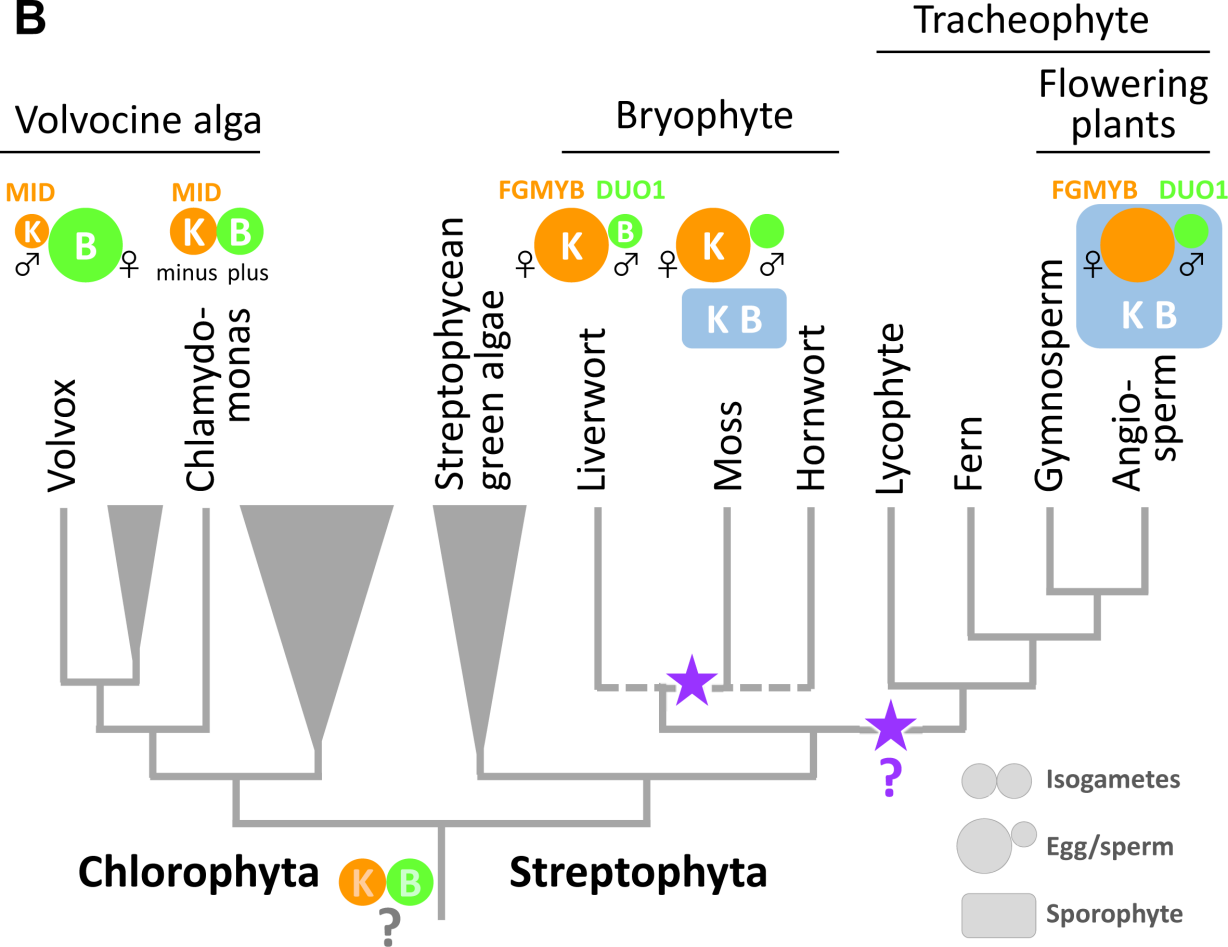# Correction: Deep evolutionary origin of gamete-directed zygote activation by KNOX/BELL transcription factors in green plants

**DOI:** 10.7554/eLife.76247

**Published:** 2021-12-15

**Authors:** Tetsuya Hisanaga, Shota Fujimoto, Yihui Cui, Katsutoshi Sato, Ryosuke Sano, Shohei Yamaoka, Takayuki Kohchi, Frédéric Berger, Keiji Nakajima

**Keywords:** Other

Hisanaga T, Fujimoto S, Cui Y, Sato K, Sano R, Yamaoka S, Kohchi T, Berger F, Nakajima K. 2021. Deep evolutionary origin of gamete-directed zygote activation by KNOX/BELL transcription factors in green plants. *eLife* **10**:e57090. doi: 10.7554/eLife.57090

Published 28 September 2021

We overlooked an important publication by Ortiz-Ramírez et al. (2017) implicating a role of a *BELL* family gene in sporophyte and spore formation in the moss *Physcomitrium patens*. Two more publications suggesting roles of *Physcomitium BELL* genes in sporophyte development and egg size regulation have been cited, but not highlighted well in our publication. We apologize for the lack of proper citation of the previous works.

The new citation:

Ortiz-Ramírez, C., Michard, E., Simon, A. A., Damineli, D. S. C., Hernández-Coronado, M., Becker, J. D. & Feijó, J. A. 2017. GLUTAMATE RECEPTOR-LIKE channels are essential for chemotaxis and reproduction in mosses. *Nature* 549: 91–95. DOI: 10.1038/nature23478, PMID: 28737761

#1 (Abstract)

Original text:

By contrast, in land plants such as *Physcomitrium patens* and *Arabidopsis thaliana*, KNOX and BELL proteins function in meristem maintenance and organogenesis during the later stages of diploid development.

Corrected text:

By contrast, in land plants such as *Physcomitrium patens* and *Arabidopsis thaliana*, KNOX and BELL proteins function in **sporophyte and spore formation**, meristem maintenance and organogenesis during the later stages of diploid development.

#2 (Discussion)

Original text:

While our study revealed the striking conservation of KNOX/BELL functions between *M. polymorpha* and *C. reinhardtii*, this finding is somewhat unexpected from a phylogenetic viewpoint because KNOX/BELL proteins in the model bryophyte *P. patens* control sporophyte development, as do KNOX/BELL proteins in angiosperms.

Corrected text:

While our study revealed the striking conservation of KNOX/BELL functions between *M. polymorpha* and *C. reinhardtii*, this finding is somewhat unexpected from a phylogenetic viewpoint because KNOX/BELL proteins in the model bryophyte *P. patens* control sporophyte development, as do KNOX/BELL proteins in angiosperms**, as well as egg size (Sakakibara et al., 2008, Horst et al., 2016, Ortiz-Ramírez et al., 2017**).

#3 (Figure)

In the published article, expression of a *BELL* gene in *P. patens* eggs, reported by Horst et al., 2016, was not reflected in Figure. 7B. The corrected Figure 7B is shown now.

The corrected Figure 7B is shown here:

**Figure 1:**
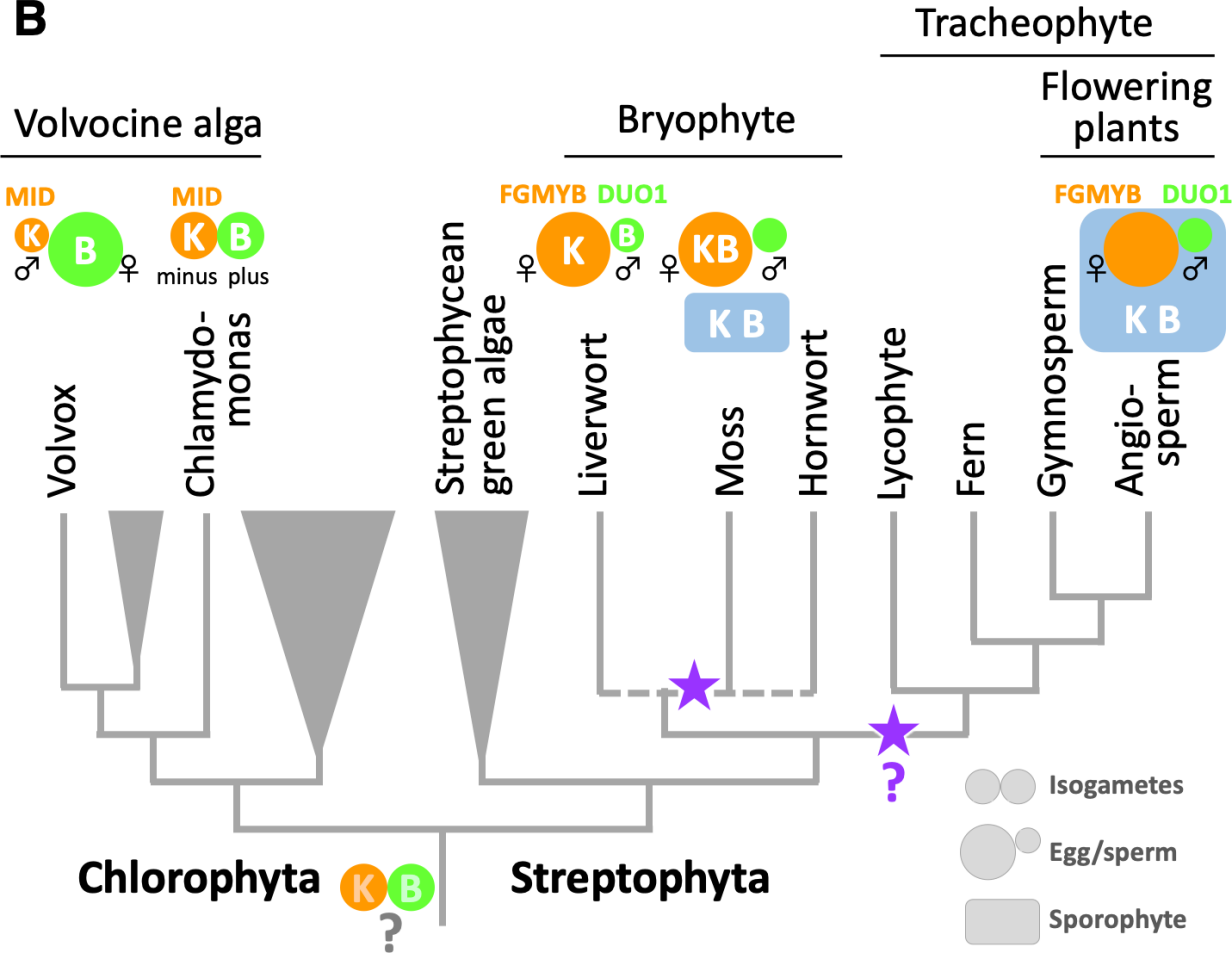


The originally published Figure 7B is also shown for reference:

**Figure 2:**